# Effect of Initial Rolling Temperature on the Microstructure Evolution of Liquified Nature Gas Low-Temperature-Resistant Steel Bars

**DOI:** 10.3390/ma18030716

**Published:** 2025-02-06

**Authors:** Zhenghong Ma, Jun Cao, Zhibo Zhang, Huanhuan Zhang, Shubiao Yin, Bingguo Liu, Xiaosong Zhang

**Affiliations:** 1 Faculty of Metallurgy and Energy Engineering, Kunming University of Science and Technology, Kunming 650093, China; mzh1070@163.com (Z.M.); jcao_cisri@foxmail.com (J.C.); zhanghuanhuan@stu.kust.edu.cn (H.Z.); zhangxiaosong@stu.kust.edu.cn (X.Z.); 2 Jiangsu Yonggang Group Co., Ltd., Suzhou 215000, China; 3 School of Mechanical and Electrical Engineering, Yunnan Open University, Kunming 650500, China; zhangzhibo100@126.com

**Keywords:** LNG low-temperature-resistant steel bar, thermal expansion curve, CCT curve, tissue phase transition, pre-eutectic reticulated ferrite

## Abstract

In order to gain insight into the changes of the organization and hardness of 500 MPa steel-grade low-temperature-resistant steel bars (HRB500DW) for liquefied nature gas (LNG) storage tanks during the continuous cooling phase transformation process, the effects of different rolling temperatures and cooling speeds on the organization of the phase change law, microstructure and hardness were studied. The results show that the critical phase transformation points A_C1_ and A_C3_ of the test steel were 702 and 880 °C, respectively. The organization of the test steel was polygonal ferrite and pearlite when the cooling rate was 1–2 °C/s. At a cooling speed of 5 °C/s, a small amount of bainite started to be produced in the region of a large deformation of rolling, and at 15 °C/s, some slate martensite started to be produced. At a cooling speed of 10 to 25 °C/s, the organization was mainly bainite. At a cooling rate of 40 °C/s, continuous pre-eutectic reticulated ferrite was formed at the austenite grain boundaries, reducing material properties. As the cooling speed increased, the hardness of the matrix organization of the test bars increased. The lower initial rolling temperature led to the expansion of the martensitic transformation zone. For rebar producers, the initial rolling temperature of 1050 °C was better than the initial rolling temperature of 1000 °C.

## 1. Introduction

In recent years, with the rapid development of the Chinese economy, energy is an important material foundation, and energy security is an important part of national resources and economic security. With the rapid development of the Chinese economy and the acceleration of the energy transition process, the proportion of LNG in the Chinese energy consumption structure has begun to rise steadily [1]. LNG has received great support and high attention from the government and oil companies during its development [2]. Additionally, it is LNG that people use to cook and heat their homes. LNG is also used as a fuel to generate electricity [3]. LNG is a liquid state formed when LNG is cooled to very low temperatures. The temperature of LNG is usually around −163 °C, which is the temperature required in order to cool LNG to its liquid state [4]. The use scenario of a low-temperature-resistant steel bar is on the outer tank and rigid foundation of the LNG storage tank, and the ambient temperature is −163 °C, which requires the concrete outer tank to be able to resist general impact and high compressive and tensile properties [5,6], and meet the strength and low-temperature toughness matching in the ultra-low-temperature environment [7,8]. Increasing the Ni content can reduce the brittleness transition temperature of steel and improve the low-temperature toughness [9]. As outlined by the national standard YB/T4641-2018, there are clear performance requirements for HRB500DW. Steel materials may be affected to different degrees in low-temperature environments, such as brittle fracture and strength reduction. The lower the temperature, the more prone to brittle fracture the material [10]. The 500 MPa steel-grade LNG HRB500DW designed in this project was used in ultra-low-temperature environments with an ambient temperature of −163 °C, where the low-temperature toughness and brittleness transition temperatures of the materials were controlled extremely strictly.

At present, foreign HRB500DW manufacturers include ArcelorMittal (Luxembourg, process: use of post-roll quenching and self-tempering technology), Sumitomo Metals (process: the use of the TMCP process to produce low-carbon niobium-containing low-temperature steel bars), etc. Among them, ArcelorMittal is in the leading position, with a history of more than 30 years of HRB500DW production and more than 300 LNG tank projects around the world being adopted. Domestic manufacturers with a large-scale production capacity of low-temperature-resistant rebar include Ma Steel, Nanjing Iron and Steel Group Co., Ltd. Steel and TISCO, with a production capacity of 2000–4000 t/a. The low-temperature-resistant rebar produced by Ansteel Steel has been applied in Sinopec’s Guangxi and Tianjin LNG projects domestically and Russia’s Arctic LNG projects abroad, while Nanjing Iron and Steel Group Co., Ltd. Steel has researched, developed and produced a special sleeve for low-temperature-resistant ribbed rebar [11]. The production level of Chinese low-temperature-resistant rebar is comparable to that of the top level. There is still a gap between the Chinese low-temperature-resistant ribbed rebar production level and the top level of the world. In 2014, Ansteel Steel Industry piloted the production of low-temperature rebar, combined with the KRYBAR low-temperature rebar production process, using low-temperature rebar through the water cooling process. In order to ensure the rebar has high strength and fracture toughness under low-temperature conditions, through the rapid water piercing cooling in the continuous rolling process, the thickness of the martensitic ring is 2 mm, and the heart is less affected by the water piercing, which is ferrite and pearlite. After the final rolling process, the temperature in the core radiates to the surface of the rebar, creating a reverse red tempering condition. This leads to the transformation of the martensite ring (MR) into the soxhite ring (SR), resulting in minimal strength loss for the rebar while significantly enhancing its toughness. The tempered martensite ring and the ferrite and pearlite cores play a major role in tensile strength and ductility, respectively, to achieve the purpose of improving the toughness of the rebar [12].

During the production of high-performance rebars, various parameters in the process have an impact on the microstructure and mechanical properties of rebars, and the core technology is the reasonable matching of the parameters of the controlled rolling and cooling process [13,14]. Controlled accelerated cooling after rolling produces a fine final microstructure and facilitates the formation of low-transition-temperature products, such as bainite and martensite/austenite (M/A) compositions. The initial rolling temperature of the cast billets for bars and wires is one of the key indicators. It reflects the initial rolling temperature state of the profile. The rolling temperature has a significant impact on grain refinement and precipitation characteristics, playing a decisive role in mechanical properties. At the same time, during the initial rolling process, the deformation of the steel billet is at its maximum. If not properly controlled, the steel billet is prone to surface cracks and may also lead to internal defects, such as delamination and inclusions. Rolling in the region from the austenite recrystallization zone to the austenite non-recrystallization zone can provide ideal microstructural characteristics for impact properties and strength by inducing a uniformly fine-grain structure, well-developed dislocation structure within the grains, and a relatively high density of fine carbides. When developing a new steel grade, the determination and study of the CCT curve is an important basic task. In particular, for the same chemical composition studied in this paper, two microstructures were formed by controlled rolling and cooling technologies. Therefore, the dynamic CCT curves of low-temperature-resistant steel HRB500DW at different rolling temperatures were studied. Therefore, this paper investigates the dynamic CCT curves of HRB500DW at different initial rolling temperatures. The calculation model of software JMatPro version 7.0 is applicable to both carbon and low-alloy steels [15]. The phase transformation law of LNG HRB500DW is summarized, and a more suitable rolling parameter was searched for to improve the production process in the steel mill.

## 2. Materials and Methods

**Figure 1:**
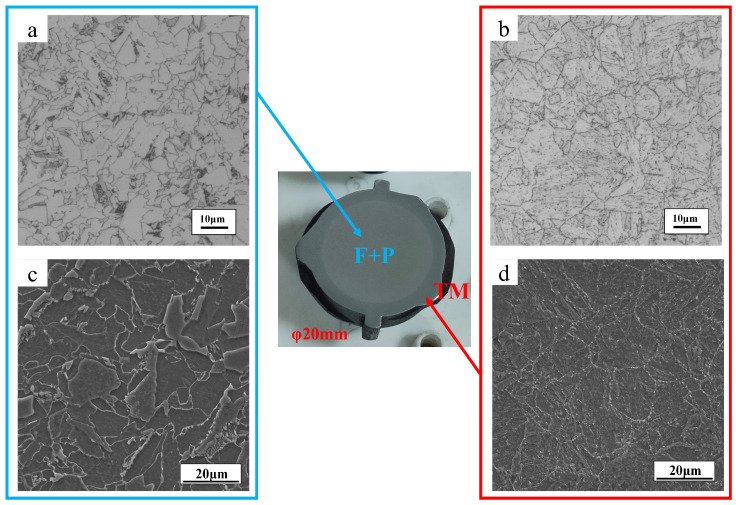
Matrix organization of HRB500DW: (**a**) core OM; (**b**) border OM; (**c**) core SEM; (**d**) border SEM.

The test raw materials were all hot-rolled ribbed steel bars with a diameter of 20 mm, and their chemical compositions are shown in Table 1. The equilibrium phase calculation of the material with JMatPro software, CCT curve calculation, and various matrix may appear in the precipitation phase composition, with the change in temperature relationship. The organization of the test raw materials is shown in Figure 1, and the materials used for thermal simulation are all taken from the center part of Figure 1. The structure of the test raw materials is shown in Figure 1, the core structure of the low-temperature-resistant steel bar is ferrite and pearlite (F + P), and the edge structure is tempered martensite (TM), also called Soxenite. The materials used in the thermal simulations are shown in the center of Figure 1.

**Figure 2:**
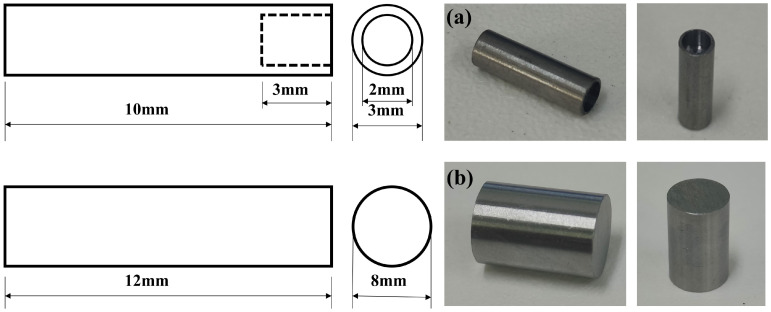
Specimen dimensions: (**a**) thermal expansion specimen; (**b**) thermal simulation specimen.

**Figure 3:**
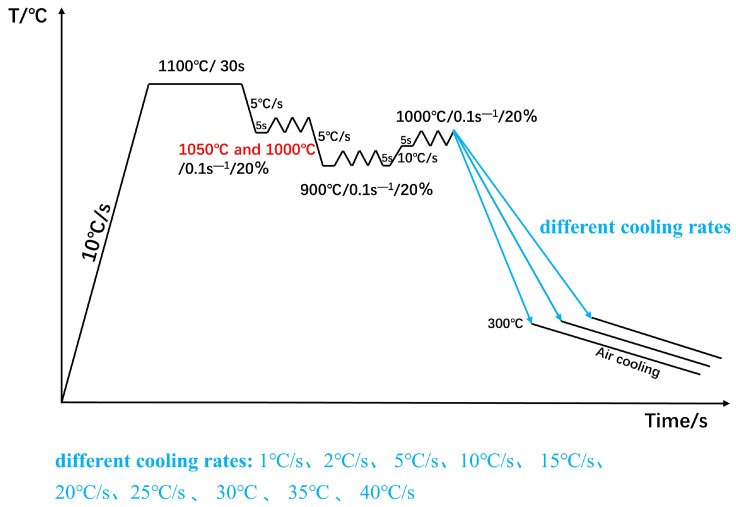
The thermal simulation test process of the test steel.

The thermal expansion specimen was processed into the specimen shown in Figure 2, placed in the thermal expansion apparatus, heated up to 1000 °C at a rate of 200 °C/h, held for 20 min, the thermal expansion curve was measured, and the critical transition temperatures A_C1_ and A_C3_ were determined by the curve. The dynamic CCT specimen was processed into a cylinder with a diameter of 8 mm and a height of 12 mm, and the surface finish of the cylinder was 1.6. Thermal simulation tests on the THERMECMASTOR thermal simulation tester (Fuji Denwave Koki Co., Ltd., Tokyo, Japan), the specific test process was as follows: the specimen was heated at a rate of 10 °C/s to 1100 °C insulation 30 s, after the rate of 5 °C/s cooled to a different initial rolling temperature, with an insulation of 5 s, deformation of 20%, deformation rate of 1 s^−1^, DW5 experimental group of the initial rolling temperature was 1000 °C and the initial rolling temperature of the experimental group DW6 rolling temperature was 1050 °C, simulating different rough rolling temperatures. The deformed specimens were then cooled at a rate of 5 °C/s to 900 °C for finish-rolling, held for 5 s, and then deformed by 20% with a deformation rate of 1 s^−1^ to simulate the finish-rolling process. After finish-rolling, the actual produced bars would briefly warm up the material due to the large deformation rolling in finish-rolling. After finishing rolling, the temperature was simulated to be increased to 1000 °C at a heating rate of 10 °C/s, held for 5 s, and deformed by 20% at a deformation rate of 1 s^−1^ to simulate the final rolling process. The specimens were then cooled to 300 °C at the cooling rates of 1, 2, 5, 10, 15, 20, 25, 30, 35, and 40 °C/s, respectively, and then air-cooled at room temperature to simulate the self-tempering process of the steel bars after water penetration. DW5 and DW6 were used as the test controls to simulate the phase transformation of the matrix organization for different initial rolling temperatures at different cooling rates. Figure 3 shows the thermal simulation scheme.

After the thermal expansion test was completed, metallographic specimens were prepared by cutting off the middle deformed section of the specimens and cutting from the center along the axial direction. The surface of the specimen was ground and polished, and the surface of the sample was corroded with a 4% volume fraction of nitric acid alcohol solution, and then the microstructure of the specimen was observed with a GX51-type optical microscope (OM) and an EVO250 scanning electron microscope (SEM) at different cooling speeds. The microhardness value of the deformed organization was determined by using the HV-1000 type Vickers hardness tester with a loading load of 300 N and a loading time of 15 s, and 12 points were measured for each specimen, and the average value was taken after removing the maximum and minimum values. Combining the metallographic method, the hardness method and phase transformation temperature, we can plot the dynamic CCT curve of the material.

## 3. Experimental Conclusions and Analysis

### 3.1. Theoretical Calculation of the Static CCT Curve

**Figure 4:**
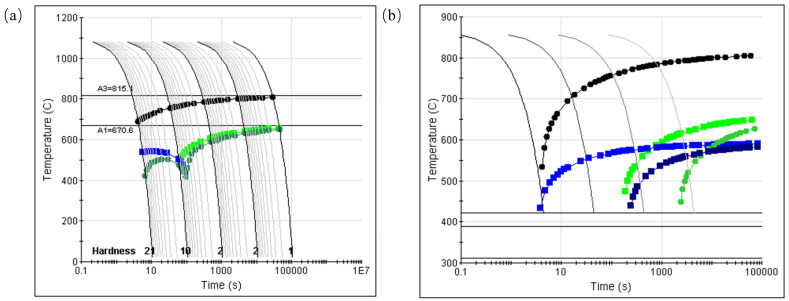
JMatPro calculation of static CCT for HRB500DW: (**a**) advanced CCT and (**b**) ordinary CCT.

The continuous cooling transition curve of the low-temperature-resistant steel bar HRB500DW was calculated by JMatPro simulation software, the static CCT curve was calculated, and the possible precipitate phase composition and temperature in the matrix were analyzed. As shown in Figure 4a of the advanced CCT simulation results, it had a higher cooling rate, from which A_C1_ = 670.6 °C and A_C3_ = 815.1 °C, the microstructure began to change the cooling velocity, and the theoretical calculation of the low-temperature-resistant steel bar generated ferrite at any cooling rate with the increase in the cooling rate. As the temperature of the austenite-to-ferrite transition decreased, the temperature point of the pearlite transition also decreased, but the bainite transition temperature increased with the increase in the cooling rate. Bainite is formed at 7 °C/s and martensite at 15 °C/s. From Figure 4b showing an ordinary CCT, it can be read that the tissues began to transition at temperature points P_s_ = 670.5 °C, B_s_ = 592.3 °C, M_s_ = 421.4 °C, and M_f_ = 312.2 °C. Fs represents the temperature at which ferrite begins to be generated, P_s_ represents the temperature at which pearlite begins to be generated, B_s_ represents the temperature at which bainite begins to be generated, M_s_ represents the temperature at which martensite begins to be generated, and M_f_ represents the temperature at which the martensite transformation is completed. A_C1_ represents the temperature at which ferrite begins to transition to austenite, and A_C3_ represents the temperature at which the austenitic adaptation is complete.

**Figure 5:**
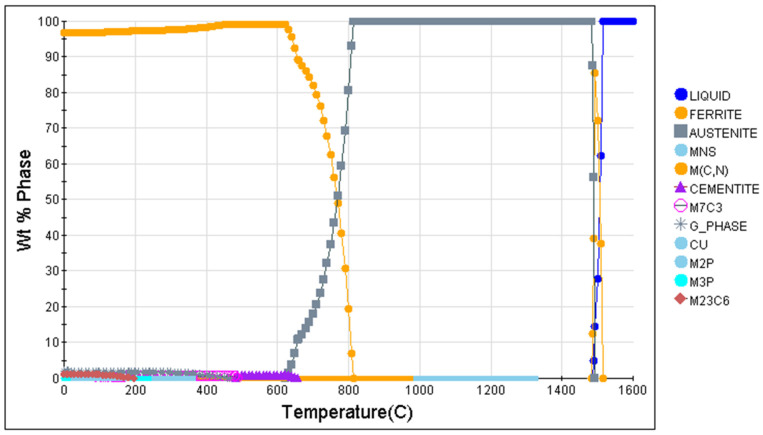
Equilibrium phase diagram for the JMatPro calculation of HRB500DW.

**Figure 6:**
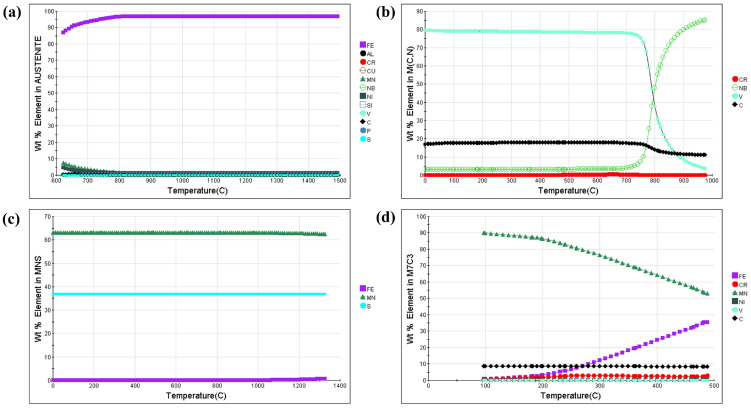

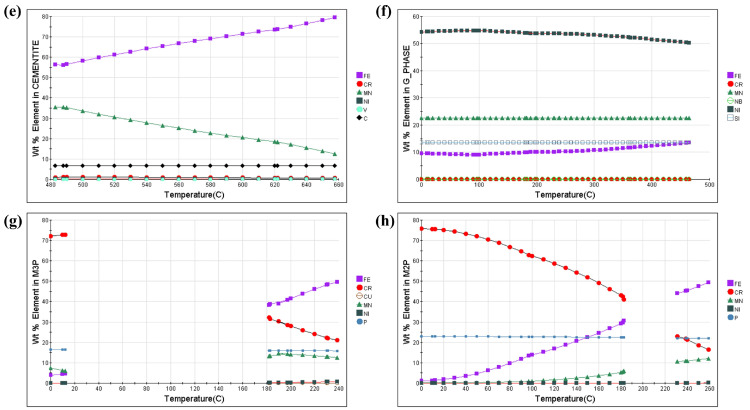
JMatPro calculates the variation rule for the precipitated-phase composition with the temperature for HRB500DW: (**a**) Austenite; (**b**) M(C, N); (**c**) MnS; (**d**) M_7_C_3_; (**e**) Cementite; (**f**) G phase; (**g**) M_3_P; (**h**) M_2_P.

Figure 5 shows the JMatPro calculation equilibrium phase diagram of HRB500DW, and from the results of the calculation, it can be seen that the HRB500DW of ferrite at 671 °C begins austenitization, and at 815 °C completes austenitization. On the basis of the equilibrium diagram, the various types of the precipitation phase composition with the trend of temperature change were analyzed, as seen in Figure 6, which shows the precipitation phase composition with the change rule in temperature. In the process of the austenite-to-ferrite transformation, the Fe element content in austenite decreases, and the Mn and Ni elements rise. Finally, in the ferrite at 621 °C, the mass ratio of the Fe element is 87.07%, the mass ratio of the Mn element is 7.62%, and the mass ratio of the Ni element is 4.49%. After the austenite-to-ferrite transformation is over, the overall ferrite content in the matrix decreases as the temperature continues to decrease because below 600 °C the matrix precipitates second-phase particles that consume a portion of the ferrite. When the temperature drops to a critical value, V and Nb precipitate to form the second-phase particles MC/MN/M(C, N), which can effectively peg grain boundaries and dislocations, and play a role in grain refinement and precipitation strengthening [16,17]. HRB500DW begins to generate M(C, N) below 975 °C, and the content of the alloying element V in M(C, N) increases as the temperature decreases, with the equilibrium state of room-temperature V mass accounting for 79.69%, and the content of the alloying element Nb gradually decreasing, with Nb mass accounting for 3.25% in the room-temperature equilibrium state. It can be deduced that the alloying element V in the HRB500DW enters into the M(C, N) precipitation particles in large quantities, which mainly produces the effect of second-phase particle precipitation strengthening, while the alloying element Nb enters into the matrix organization in large quantities, which mainly produces the effect of solid solution strengthening.

At 488 °C below the beginning of the generation, with the lowering of the temperature, M_7_C_3_ in the Mn element will increase and the proportion of Fe elements will decline. Moreover, the room-temperature Mn element mass proportion will be 90%, C element mass proportion 8.57%, and the remaining will be the alloying elements. Therefore, by adding the V and Nb elements, both precipitation strengthening and solid solution strengthening play a role in strengthening the matrix. This is also a means to simultaneously ensure the matching of material strength and low-temperature toughness. Carbide begins to be generated below 657 °C. As the temperature decreases, the Mn element in the carbide will increase, the proportion of the Fe element will decrease, and the mass proportion of the Mn element at room temperature will be 35.55%, the mass proportion of the Fe element at room temperature 56.45%, the content of the C element in the carbide stays unchanged with a mass proportion of 6.73%, and the rest are the alloying elements, with carbide in a trace presence.

Regarding the control of the harmful nonmetallic elements sulfur and phosphorus, MnS starts to form at 1325 °C and the elemental mass ratio remains approximately the same, with 63.14% manganese, 36.86% ferrum, and a very low S elemental mass ratio. As the steel bar contains Mn elements, it is easy to form MnS inclusions with S elements. Due to the special solidification characteristics and elemental segregation behavior [18], these sulfide inclusions first have the ability to destroy the continuity of the steel base and affect the mechanical properties of duplex steel materials. Secondly, the presence of MnS particles causes the material to exhibit brittle fracture and promotes material failure [19]. At hot rolling temperatures, MnS is softer than the surrounding steel matrix and therefore tends to be elongated during the hot rolling process. These elongated inclusions affect the ductility when subjected to transverse tensile testing, leading to in-plane anisotropy in ductility [20]. Therefore, the elemental S content should be strictly controlled in HRB500DW. Phosphorus, as an impurity element, reduces the toughness of high-strength steels and exacerbates the brittle fracture. Even a very low volume concentration can lead to a severe enrichment of precursor austenite grain boundaries [21]. The precipitation of phosphide increases the cold embrittlement of steel and is a harmful element for low-temperature-resistant toughness, and the degree of grain boundary embrittlement caused by phosphorus elemental segregation is assessed according to the change in the toughness–brittle transition temperature (DBTT); the higher the amount of segregation, the more severe the embrittlement is, so there has been a consistent effort to reduce the P content in steelmaking [22]. The P content of HRB500DW is also a significant contributor to the high-temperature toughness of high-strength steel. The HRB500DW studied in this experiment maintained the sulfur element content below 20 ppm and the phosphorus element content at 70 ppm.

### 3.2. Determination of Dynamic CCT Curves

#### 3.2.1. Thermal Expansion Curve

**Figure 7:**
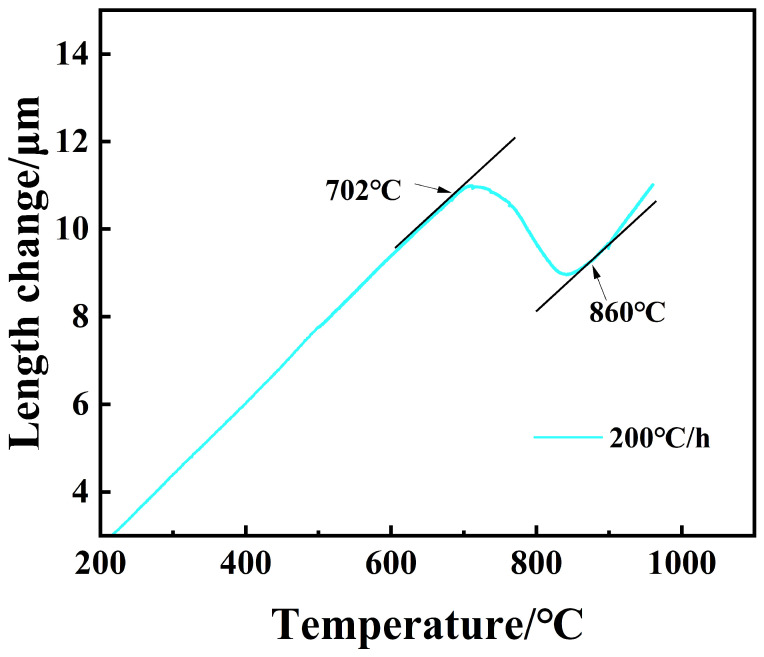
Measuring the actual A_C1_ and A_C3_ thermal expansion curves.

When the processed sample was heated at an elevated temperature, the temperature at which austenite began to form, Ac_1_, and the temperature at which complete austenitization occurred, Ac_3_, were measured. After conducting a slow heating rate of 200 °C/h for the sample in Figure 2, the results of the elevated thermal expansion curve are shown in Figure 7, which shows that the steel is heated slowly at the time of heating, with the temperature at which complete austenitization occurs, i.e., Ac_3_ = 860 °C, and the temperature at which the austenite begins to transform, i.e., Ac_1_ = 702 °C. As is shown in Table 2, it is the thermal expansion rate of the material at the key temperature turning point, which can be found that the thermal expansion rate of the room temperature structure is 1.63 × 10^−2^ μm/°C, the thermal expansion rate of the austenite structure is 1.86 × 10^−2^ μm/°C, and between the AC1 and AC3 temperatures, there are both room temperature and austenitizing structures, resulting in the change of the thermal expansion rate of the material, we take the average thermal expansion rate of −2.21 × 10^−2^ μm/°C. It should be noted that the negative thermal expansion rate here is caused by the thermal expansion of the material during the heating process, and the microstructure phase transformation occurs at the same time. The thermal expansion curve was then determined by the temperature at which the steel began to change. There are two methods for determining the critical-phase transition point during heating up or cooling down by means of thermal expansion curves, namely the vertex method and the tangent method. The vertex method utilizes the obvious inflection point as the critical point of the phase transition; the advantage of the vertex method is the ease in determining the critical point, and the disadvantage is that at the apex of the curve, it corresponds to the temperature of the occurrence of a large number of phase transitions, but it is, in fact, just off the slope of the curve, that is, the tangent point of the curve for the actual occurrence of the beginning of the phase transition temperature. Therefore, it can be seen that the starting temperature of the phase transition determined by the vertex method is lower than the actual starting temperature of the phase transition, and the ending temperature is higher than the actual temperature. The tangent method is to draw a straight line along the expansion curve and take the tangent point as the critical point of a phase change; the critical point determined by this method is closer to the actual phase change point, and the disadvantage of this method is that the separating point is arbitrary and subject to the influence of man, so the error is larger. However, the vertex method is closer to the actual onset and end temperatures of the phase change; therefore, for the continuous cooling curve, the tangent method is generally used, and the tangent method was used for the present experiment. We determined the phase transition point. It should be noted that in the process of heating or cooling when the thermal expansion curve may exist at more than one phase transition point, the temperature of the starting point of each phase transition is based on the inflection point of the curve or the slope of the sudden change to determine. However, for phase-change points or inflection points of the curve that are not obvious, it is necessary for it to be based on the cooling of the metallographic organization changes or surface hardness to assist in the identification. There are also some less obvious curve inflection points, where only a general temperature range of the phase change can be obtained. The curve inflection point is not obvious mainly because of the occurrence of the phase-change rate of the thermal expansion difference is small or the occurrence of the phase change of the fraction is small, resulting in the sample itself not being a big change in volume; hence, the equipment cannot be accurately measured.

**Figure 8:**
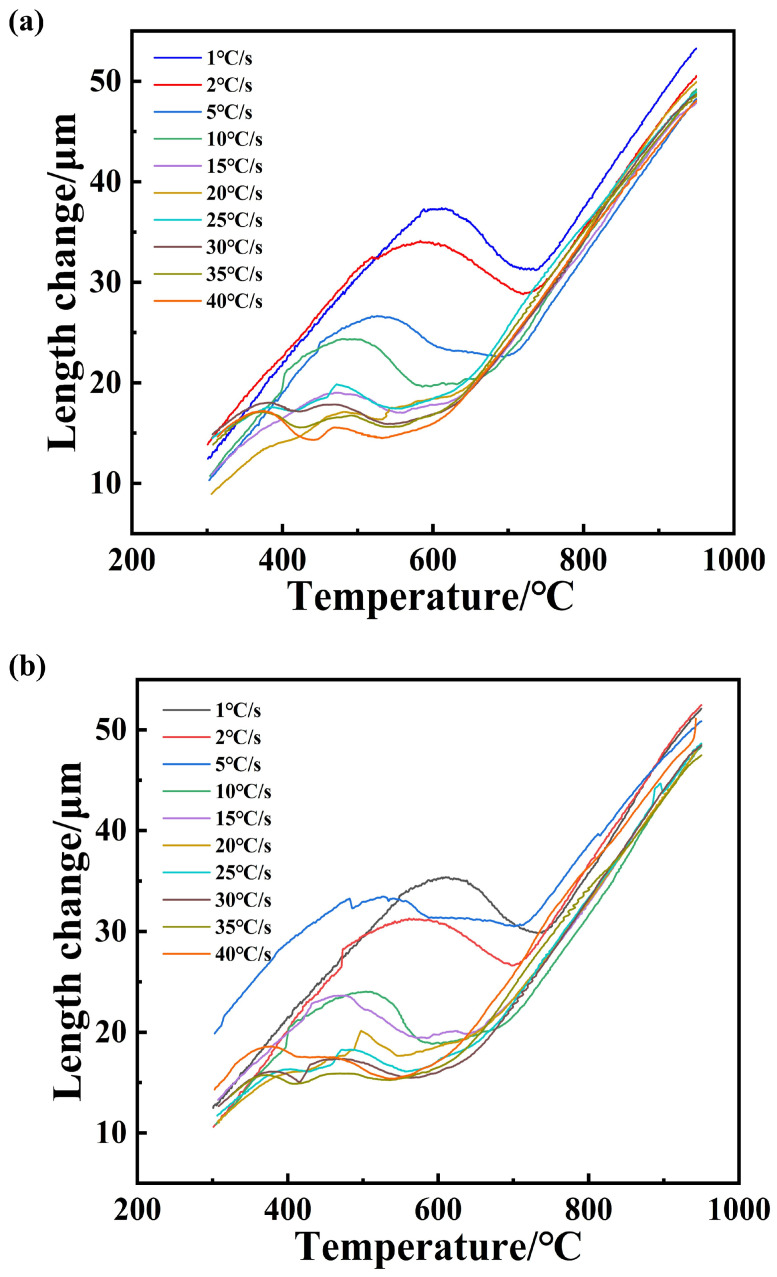
Thermal expansion curve of HRB500DW during the cooling process: (**a**) DW5 initial rolling temperature 1050 °C; (**b**) DW6 initial rolling temperature 1000 °C.

After the processed sample has been completely austenitized, in the continuous cooling process, the steel began to phase-change, the specimen expansion deviated from the linear rule of the change in thermal expansion and contraction according to the temperature–expansion curve shown in Figure 7. In the cooling process of HRB500DW, the tangent method was used to determine the start temperature of the phase change. The tangent point of the specific value of the readings had a large human error, and the error was acceptable within the ±5 °C range. This specific type of phase transition needs to be combined with microstructure and hardness to be determined. Figure 8 shows HRB500DW after the final rolling at a temperature from 1000 °C to 300 °C with different cooling down rates to the real thermal expansion curve. Table 3 summarizes the phase transition points at each cooling rate during the cooling down process after complete austenitization of the HRB500DW.

#### 3.2.2. Microstructure

**Figure 9:**
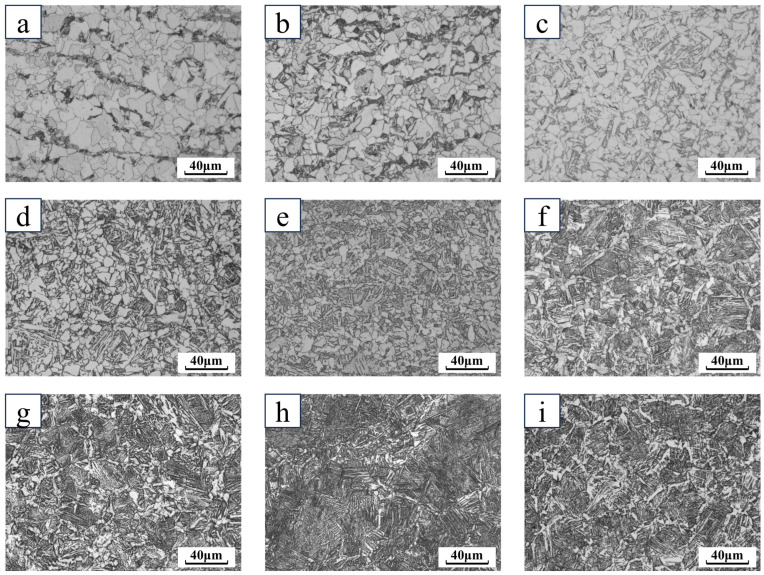
Metallographic diagram of DW5 at different cooling speeds: (**a**) 1 °C/s; (**b**) 5 °C/s; (**c**) 10 °C/s; (**d**) 15 °C/s; (**e**) 20 °C/s; (**f**) 25 °C/s; (**g**) 30 °C/s; (**h**) 35 °C/s; (**i**) 40 °C/s.

**Figure 10:**
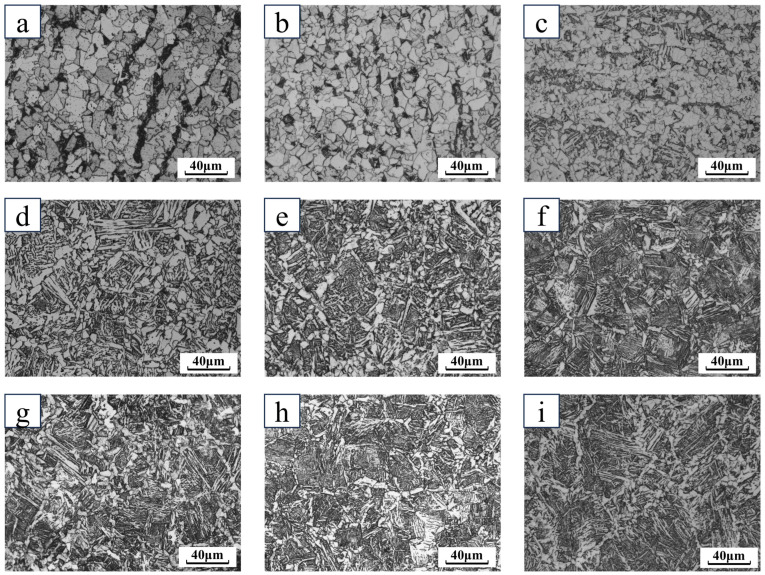
Metallographic diagram of DW6 at different cooling speeds: (**a**) 1 °C/s; (**b**) 5 °C/s; (**c**) 10 °C/s; (**d**) 15 °C/s; (**e**) 20 °C/s; (**f**) 25 °C/s; (**g**) 30 °C/s; (**h**) 35 °C/s; (**i**) 40 °C/s.

As can be seen from Figure 9 and Figure 10, when the cooling rate was 1 °C/s and 2 °C/s, the microstructure consisted of white ferrite and black–brown pearlite, and when the cooling rate was increased to 5 °C/s, the bainite organization was formed, for the first time, in a small number of large deformations. The emergence of the grain size decreases with the increase in the cooling rate, and when the cooling rate is further increased, the emergence of the supercooled austenite is a high number of transformations to bainite. DW5 at a cooling rate of 15 °C/s began to generate martensite. DW6 at a cooling rate of 10 °C/s began to generate martensite. With the increase in the cooling speed, the bainite content appeared to increase and then decrease, and the turning line was at 25 °C/s. After 25 °C/s, a large amount of slat martensite was formed, and the bainite organization gradually decreased. At the same time, the grain size of slat martensite decreased. When the cooling rate reached above 35 °C/s, the organization was martensite and ferrite. At a cooling rate of 30 °C/s or more, ferrite distributed along grain boundaries appeared in both DW5 and DW6. At 30 °C/s, the ferrite precipitated along the grain boundaries is small in size and has a tendency to join into a reticulated ferrite. At 40 °C/s, the matrix organization of DW5 and DW6 is mostly martensite, and there is a small part of continuous pre-eutectic reticulated ferrite organization at the martensite grain boundaries.

**Figure 11:**
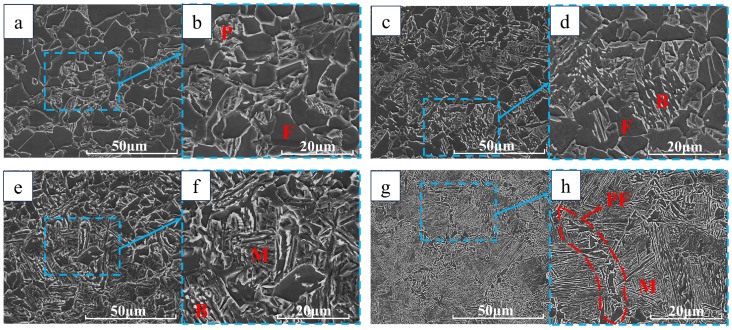
Electron microscope scans of DW5 at different cooling speeds: (**a**) 1 °C/s; (**b**) 1 °C/s; (**c**) 10 °C/s; (**d**) 10 °C/s; (**e**) 25 °C/s; (**f**) 25 °C/s; (**g**) 40 °C/s; (**h**) 40 °C/s.

**Figure 12:**
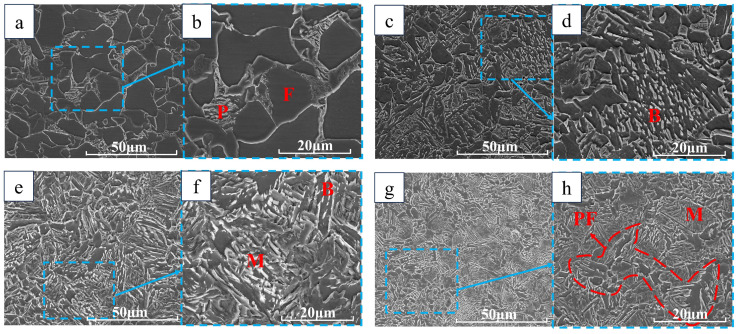
Electron microscope scans of DW6 at different cooling speeds: (**a**) 1 °C/s; (**b**) 1 °C/s; (**c**) 10 °C/s; (**d**) 10 °C/s; (**e**) 25 °C/s; (**f**) 25 °C/s; (**g**) 40 °C/s; (**h**) 40 °C/s.

In Figure 11 and Figure 12, we performed SEM on the test groups with cooling rates of 1 °C/s, 10 °C/s, 25 °C/s, and 40 °C/s. It can be seen that at a cooling rate of 1 °C/s, ferrite and pearlite lamellar structures can be seen. At 10 °C/s, the carbide precipitation of bainite is seen as discontinuous linear, the martensite carbide precipitation seen at 25 °C/s is smooth and large in size, and the martensite carbide precipitation at 40 °C/s has needle-like characteristics and the carbide is elongated. From the comparative analysis of the DW5 and DW6 test groups, we can find that at the same cooling rate, the grains of DW5 are smaller, above the cooling rate of 25 °C/s, the structure is martensite, but the carbide morphology is different, the martensite carbide precipitation at a high cooling speed will be more slender and sharp, and the martensite formed at a lower cooling speed will be more rounded and larger in size.

The morphology of pre-eutectic ferrite is mainly affected by the mass fraction of carbon, cooling rate and heating temperature. When the mass fraction of carbon is low, the main formation of massive ferrite occurs. When the chemical composition of the steel is certain, the morphology of the first eutectic ferrite is related to the cooling rate. The slower the cooling rate, the first eutectic ferrite morphology tends to block, and the higher the amount, the larger the block. The faster the cooling rate, the more pre-eutectic ferrite morphology tends to block network, and the lower the amount, the finer the network distributed along the grain boundaries. The pre-cooling time before quenching was different and the first eutectic ferrite morphology was different. Due to the effect of hardenability, from the surface layer to the center, the cooling rate went from fast to slow, and the morphology of the first eutectic ferrite from an intermittent, fine semi-reticulate to coarse reticulate transition.

#### 3.2.3. Hardness Analysis

A microhardness tester was used to measure the hardness of different microstructures in the HRB500DW in order to further clarify the type of organization, and the results of the measurements are shown in Table 4. The Vickers hardness of the matrix organization was 200 HV at a cooling rate of 1 °C/s for DW5, 207 HV at a cooling rate of 1 °C/s for DW6, 246 HV at 15 °C/s for DW5, 255 HV at 40 °C/s for DW6, and 255 HV at 40 °C/s for DW6, which was the same as that of HRB500DW. The Vickers hardness was 246 HV for DW6 at a 15 °C/s cooling rate, the matrix organization Vickers hardness was 255 HV forDW5 at a 40 °C/s cooling rate, the matrix organization Vickers hardness was 289 HV for DW6 at a 40 °C/s cooling rate, and the matrix organization Vickers hardness was 291 HV. With the increase in the cooling rate, the specimen matrix organization of the average hardness value increased. This is because the increase in the cooling rate causes the proportion of the hard-phase rate to increase, leading to an increase in the proportion of hard phases and gradual grain refinement. In addition, decreasing the initial rolling temperature increases the hardness of the matrix organization. At the same cooling rate, the hardness value of the matrix organization of the initial rolling at 1000 °C was higher than that of the initial rolling at 1050 °C. The reason is that at lower temperatures, the hardness value of the matrix organization of the initial rolling is higher than that of the initial rolling.

**Figure 13:**
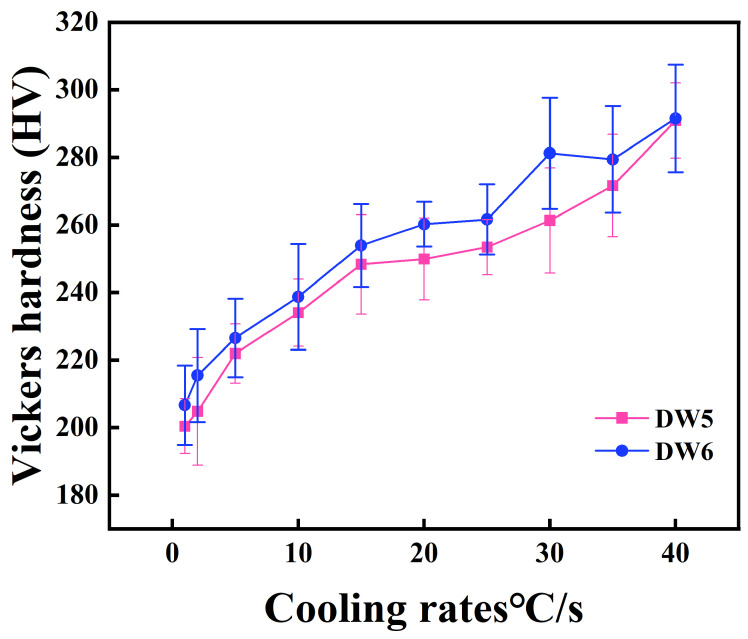
Hardness statistics for test samples DW5 and DW6.

The reason is that at lower-temperature rolling, matrix organization resistance increases, resulting in a greater density of the original austenite intracrystalline defects in the center of the rebar. Moreover, the ferrite phase transition provides more nucleation locations, resulting in the low-temperature initial rolling temperature conditions in the center of the rebar of ferrite fine and uniform, etc. [23]. The greater the cold speed, the greater the austenite nucleation rate, and the finer the austenite grain, the higher the matrix organization of the strength and hardness. The higher the cooling speed, the shorter the phase transition time controlled by the diffusion process, which limits the diffusion of carbon atoms and grain boundary movement, inhibits diffusion-type transformations such as ferrite and pearlite, and making it easier to carry out shear-type phase transitions [24], generating hard phases and improving the hardness of the matrix organization. Table 4 shows microhardness statistics under different process conditions, and Figure 13 is the hardness value line graph.

#### 3.2.4. Dynamic CCT Curve

According to the phase transition points determined by the expansion curves and the metallographic organization observation results, the organization types of the HRB500DW at different deformation temperatures and cooling rates were obtained, as shown in Table 3. The dynamic CCT curves of the HRB500DW at deformation temperatures of 1050 °C and 1000 °C were plotted.

It was found that the critical-phase transition point temperature decreased gradually with the increase in cooling rate. This is because the increase in cooling rate leads to an increase in the degree of subcooling, prompting the austenite-to-ferrite transformation of the free-enthalpy difference increases, grain boundaries, dislocations, and other places of the critical nucleation free energy and uniform nucleation critical nucleation free energy compared to the gradual decrease. Hence, the phase change is easy to carry out in the grain boundaries, and it is easier to nucleate when the grain organization is smaller.

**Figure 14:**
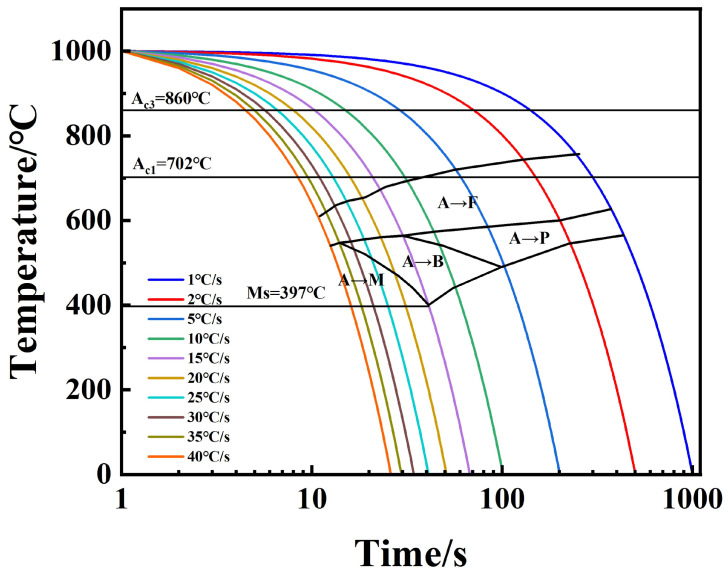
DW5 dynamic CCT thermal simulation continuous cooling curve.

**Figure 15:**
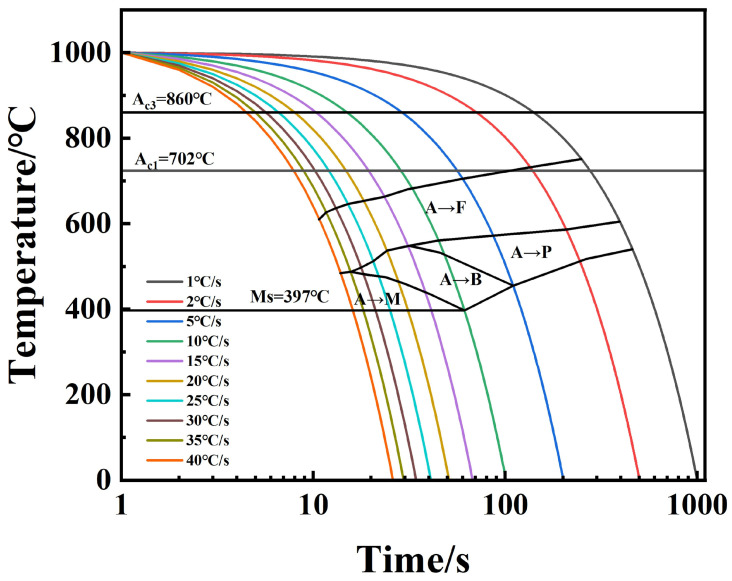
DW6 dynamic thermal simulation continuous cooling curve.

**Figure 16:**
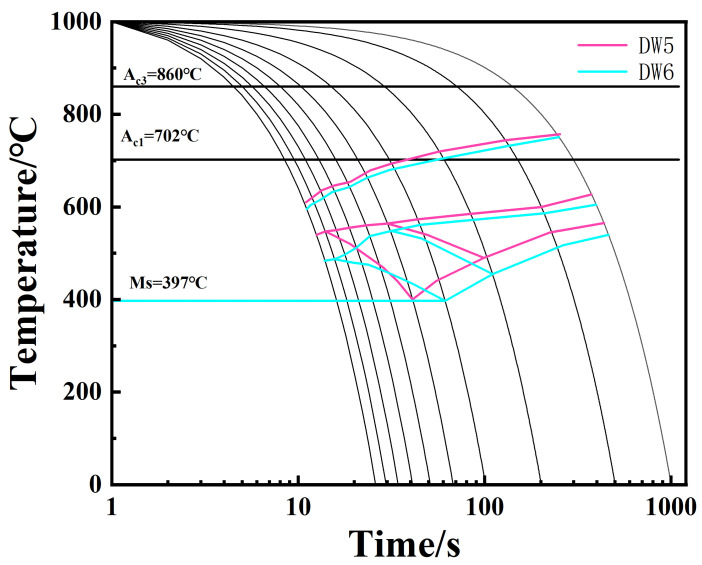
Stacked comparison of the dynamic CCT curves between DW5 and DW6.

According to the dynamic CCT organization change rule, a 1050 °C initial rolling temperature expanded ferrite and the pearlite phase transition zone, while the martensite phase transition zone slightly reduced. The high-temperature ferrite and pearlite phase transition zone expansion occurred because with high-temperature ferrite, the pearlite phase changes into the interface-controlled diffusion-type phase transition, and a high temperature is also conducive to the diffusion of the Fe and C elements. At the same time, deformation rolling leads to a large number of defects within the matrix organization. A high amount of storage for distortion energy at the same time is also conducive to the diffusion of the Fe and C elements. Carbon and nitride will precipitate at the austenite grain boundaries under the action of deformation, so that the stability of austenite is reduced, and the ferrite phase transition of the gestation period is shortened to promote the occurrence of the ferrite and pearlitic phase transition to expand the phase transition zone of [25]. At a 1000 °C initial rolling temperature, the martensite phase transition zone expanded, and DW6 at a 15 °C/s cooling rate only began to generate martensite, while DW5 at 10 °C/s began to generate martensite. Due to the large difference in the specific volume of martensite and supercooled austenite, the energy that can be provided by the common lattice interface of the two is much smaller than the resistance encountered during the martensite phase transformation, so sufficiently large supercooling is required for the phase transformation to proceed smoothly [26]. The low initial rolling temperature is not conducive to the diffusion of the Fe and C elements, and the low-temperature rolling deformation of the grain boundaries will gather more distortion energy, so that the supercooling required for the martensite is reduced in strength and it is easier to produce This hard phase martensite. Table 5 below shows the types of organizations in the HRB500DW at different deformation temperatures and cooling rates. Figure 14 and Figure 15 show the dynamic CCT curves of HRB500DW at different initial rolling temperatures. In order to facilitate the reader, the difference between the above two dynamic CCT curves was better compared. We compared the two tissue change curves in a stack. The comparison of the dynamic CCT stacks between DW5 and DW6 is shown in Figure 16.

### 3.3. Mechanical Testing

**Figure 17:**
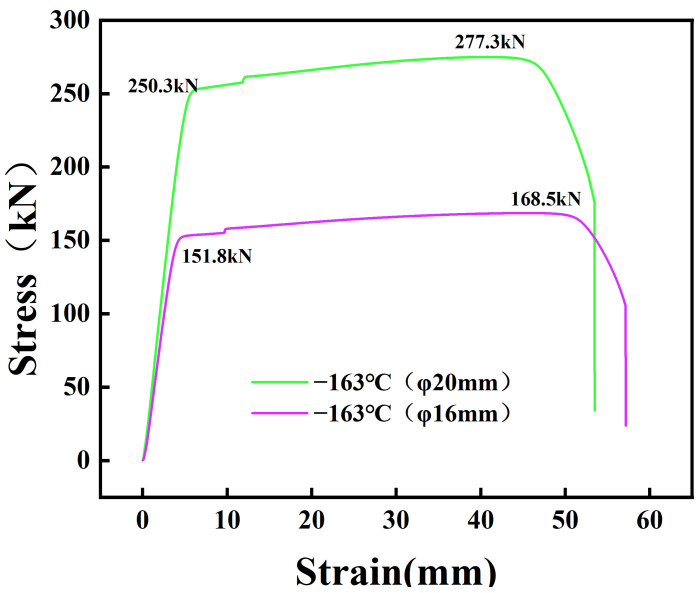
Engineering stress–strain curve.

As is shown in Figure 17, we carried out the axial tensile test at −163 °C for the low-temperature-resistant steel bars up to φ16 mm and φ20 mm, and the mechanical property results are shown in Table 6 below. YB/T 4641-2018 [27] requires that the yield strength of low-temperature-resistant steel bars at −163 °C is greater than 575 MPa, i.e., the maximum force total elongation; the non-notched sample is greater than 3%; and the notched sample is greater than 1.0%, and the results show that the performance is up to standard. R_m_ represents the yield strength of the material, RP_0.2_ represents the plastic deformation strength of the material when the material is deformed by 0.2%, A% represents the elongation at the fracture, and A_gt_% represents the elongation at the maximum force.

## 4. Conclusions

In this paper, the dynamic CCT curve of the low-temperature-resistant steel bars was drawn, and the effects of different rolling temperatures and cooling rates on its hardness, microstructure and phase transformation law were studied.

1. When the cooling rate was below 2 °C/s, the microstructure consisted of ferrite and pearlite. As the cooling rate increased to 5 °C/s, a small amount of bainite began to form at the locations of rolling deformation. At cooling rates above 30 °C/s, it was primarily martensite. The higher the cooling rate, the smaller the grain size of the martensite. When the cooling rate reached 40 °C/s, a continuous network of proeutectoid ferrite appeared at the martensite grain boundaries.
2. DW5 began to form martensite at a cooling rate of 15 °C/s, while DW6 started to form martensite at a cooling rate of 10 °C/s. The initial rolling temperature of 1000 °C expanded the martensite phase transformation zone.
3. With the increase in cooling rate, the hardness of the matrix structure also increased, and the grain size decreased. Lowering the initial rolling temperature increased the hardness of the matrix structure. At the same cooling rate, the hardness value of the matrix structure rolled at 1000 °C was higher than that rolled at 1050 °C.
4. By analyzing the room-temperature structure of 500 MPa steel-grade low-temperature-resistant steel bars, it was found that under an initial rolling temperature of 1050 °C, the matrix structure was slightly larger than that under an initial rolling temperature of 1000 °C, and the average hardness of the matrix structure decreased by 5%, along with a reduction in matrix dislocations and internal stress. From the perspective of structural strength alone, the initial rolling temperature of 1000 °C increased the strength of the matrix structure, but the increase in strength was limited. In practical terms, to achieve these strength increases, the corresponding cost is an increase in rolling difficulty, a decrease in rolling temperature, a reduction in the material’s plastic deformation capability, an increase in deformation resistance, and a heightened risk of cracking on the surface of the rebar, posing a challenge for production equipment and power consumption. Therefore, it is not cost-effective to significantly increase production costs for a limited increase in strength. The experimental conclusion is that the initial rolling temperature of 1050 °C for the DW6 group is a better choice for production.

## Figures and Tables

**Table 1:** Chemical composition of the tested steel (wt.%).

**Steel**	C	Si	Mn	Ni	V	Al	P	S	N	Fe
**HRB500DW**	0.07	0.30	1.51	1.03	0.08	0.027	0.007	0.002	0.0081	Bal.

**Table 2:** Table of the thermal expansion rate of low-temperature-resistant steel bars.

Steel	Temperature (°C)	Expansion (μm/°C)
HRB500DW	300	1.63 × 10^−2^
HRB500DW	400	1.63 × 10^−2^
HRB500DW	500	1.63 × 10^−2^
HRB500DW	600	1.63 × 10^−2^
HRB500DW	702	1.63 × 10^−2^
HRB500DW	750	−2.21 × 10^−2^
HRB500DW	800	−2.21 × 10^−2^
HRB500DW	860	1.86 × 10^−2^
HRB500DW	900	1.86 × 10^−2^
HRB500DW	950	1.86 × 10^−2^

**Table 3:** Phase transition temperature points at each cooling rate of DW5 and DW6.

Initial Rolling Temperature	Cooling Rate (°C/s)	F_s_/°C	P_s_/°C	B_s_/°C	M_s_/°C
DW5 (1050 °C)	1	747	627		
	2	744	600		
	5	720	585	580	
	10	693	573	570	
	15	670	564	564	397
	20	654		555	436
	25	646		550	471
	30	636		547	500
	35	627		541	523
	40	610			547
DW6 (1000 °C)	1	751	605		
	2	732	586		
	5	704	572	568	
	10	680	561	556	397
	15	663	548	548	435
	20	643		537	459
	25	632		512	475
	30	615		497	492
	35	605		487	512
	40	589			530

**Table 4:** Microhardness under different process conditions.

Initial Rolling Temperature	Cooling Rate (°C/s)	HV	Initial Rolling Temperature	Cooling Rate (°C/s)	HV
DW5 (1050 °C)	1	200	DW6 (1000 °C)	1	207
	2	203		2	217
	5	224		5	227
	10	243		10	237
	15	246		15	255
	20	245		20	260
	25	250		25	261
	30	258		30	279
	35	275		35	281
	40	289		40	291

**Table 5:** Types of organizations under different process conditions.

Initial Rolling Temperature	Cooling Rate (°C/s)	Metal Organization	Initial Rolling Temperature	Cooling Rate (°C/s)	Metal Organization
DW5 (1050 °C)	1	F + P	DW6 (1000 °C)	1	F + P
	2	F + P		2	F + P
	5	F + P + B		5	F + P + B
	10	F + P + B		10	F + P + M
	15	F + B + M		15	F + B + M
	20	F + B + M		20	F + B + M
	25	F + B + M		25	F + B + M
	30	F + B + M		30	F + B + M
	35	F + B + M		35	F + B + M
	40	F + M		40	F + M

**Table 6:** Results of tensile mechanical properties at low temperatures.

Steel	φ	R_m_/MPa	RP_0.2_/MPa	R_m_/RP_0.2_	A%	A_gt_%	Tensile Temperature	Notch
HRB500DW	20 mm	883	796	1.11	16	8.64	−163 °C	no
HRB500DW	16 mm	838	751	1.12	14	6.21	−163 °C	no

## Data Availability

The raw data supporting the conclusions of this article will be made available by the authors on request due to privacy.
